# T Cells Actively Infiltrate the White Matter of the Aging Monkey Brain in Relation to Increased Microglial Reactivity and Cognitive Decline

**DOI:** 10.3389/fimmu.2021.607691

**Published:** 2021-02-16

**Authors:** Katelyn V. Batterman, Payton E. Cabrera, Tara L. Moore, Douglas L. Rosene

**Affiliations:** ^1^Laboratory for Cognitive Neurobiology, Department of Anatomy & Neurobiology, Boston University School of Medicine, Boston, MA, United States; ^2^Laboratory of Interventions for Cortical Injury and Cognitive Decline, Department of Anatomy & Neurobiology, Boston University School of Medicine, Boston, MA, United States; ^3^Center for Systems Neuroscience, Boston University, Boston, MA, United States

**Keywords:** myelin, T cells, aging, cognitive decline, microglia, blood brain barrier, white matter

## Abstract

Normal aging is characterized by declines in processing speed, learning, memory, and executive function even in the absence of neurodegenerative diseases such as Alzheimer's Disease (AD). In normal aging monkeys and humans, neuronal loss does not account for cognitive impairment. Instead, loss of white matter volume and an accumulation of myelin sheath pathology begins in middle age and is associated with cognitive decline. It is unknown what causes this myelin pathology, but it likely involves increased neuroinflammation in white matter and failures in oligodendrocyte function (maturation and repair). In frontal white matter tracts vulnerable to myelin damage, microglia become chronically reactive and secrete harmful pro-inflammatory cytokines. Despite being in a phagocytic state, these microglia are ineffective at phagocytosing accruing myelin debris, which directly inhibits myelin sheath repair. Here, we asked whether reported age-related increases in pro-inflammatory markers were accompanied by an adaptive immune response involving T cells. We quantified T cells with immunohistochemistry in the brains of 34 cognitively characterized monkeys and found an age-related increase in perivascular T cells that surround CNS vasculature. We found a surprising age-related increase in T cells that infiltrate the white matter parenchyma. In the cingulum bundle the percentage of these parenchymal T cells increased with age relative to those in the perivascular space. In contrast, infiltrating T cells were rarely found in surrounding gray matter regions. We assessed whether T cell infiltration correlated with fibrinogen extravasation from the vasculature as a measure of BBB leakiness and found no correlation, suggesting that T cell infiltration is not a result of passive extravasation. Importantly, the density of T cells in the cingulum bundle correlated with microglial reactivity and with cognitive impairment. This is the first demonstration that T cell infiltration of white matter is associated with cognitive decline in the normal aging monkey.

## Introduction

Even in the absence of pathologic neurodegeneration, impairments in learning, memory, executive function, and processing speed begin as early as the third decade (1) making cognitive decline a hallmark of both healthy and neurodegenerative brain aging. In addition, there is much inter-individual variability in the severity and age of onset of these cognitive changes, with some individuals aging very successfully but about 30% of individuals suffering severe cognitive impairment without meeting the criteria for Alzheimer's disease or clinical dementia (2). The cognitive decline of normal aging cannot be attributed to neuronal loss, as neuronal density remains stable across the lifespan (3). Instead, studies using MRI and tissue analyses have found that white matter volume declines with age and correlates with cognitive impairment in healthy aged adults (4–7). Notably, damage to white matter tracts does not occur equally throughout the brain as diffusion tensor imaging has shown that, in aging humans, disruption occurs first and to a greater degree in tracts of the frontal white matter as well as the cingulate bundle compared to tracts of the occipital and temporal lobes (8–12).

The rhesus monkey is a widely used model for studying normal aging because they exhibit cognitive decline in the same domains as humans and have similar proportions of successful and unsuccessful agers (13). Extensive cognitive testing in our monkey model of normal aging has demonstrated age-related impairments in learning, memory, and executive function (14). Moreover, rhesus monkeys do not develop Alzheimer's or other neurodegenerative diseases and both gray matter volume and neuron numbers are stable across the lifespan (14–17). Thus, the rhesus monkey can serve as a model for the age-related cognitive impairment of normal aging without the confounds of occult neuropathology that challenge human studies. Importantly, rhesus monkeys also have a ratio of white to gray matter similar to humans and exhibit an age-related decrease in white matter volume and increase in myelin damage which correlate with cognitive decline (17–19). Diffusion imaging in rhesus macaques confirms the same differential vulnerability of white matter tracts observed in humans, with frontal tracts exhibiting disruption while other white matter tracts such as the internal capsule remain stable with age; and these tract disturbances correlate with cognitive impairment (20). Ultrastructural examination of our monkey brains has demonstrated that white matter pathology begins with splitting of the sheath and formation of balloons filled with degenerating cytoplasm or fluid (21, 22). Such defects are present in <1% of the sheaths in young monkeys but increase 7-fold to 7–8% in the oldest monkeys and correlate with cognitive impairment (19). These ultrastructural studies have confirmed the vulnerability of frontal white matter tracts to myelin sheath damage (19, 21, 23). Additionally, this pathology is accompanied by axon degeneration, albeit at a lower rate (0.1–0.8%) (19) so that it is likely that the observed myelin pathology impairs axon conduction producing a disconnection that likely contributes to cognitive impairment (18, 22, 24).

While the exact causes of myelin pathology in aging are unknown, several different processes may play important roles. Myelin maintenance or homeostasis relies on a complex interplay of oligodendrocyte maturation, myelin plasticity, removal and clearance of myelin debris and remyelination—processes that all are negatively affected by aging (25–29). A failure of oligodendroglia precursor cells (OPCs) to mature into myelinating oligodendrocytes results in decreased remyelination in an animal model of demyelination (30) and aged rodents exhibit a decreased ability to replenish their OPC population, which may partially underlie age-related failure of myelin repair (29). Aging humans exhibit a 34% decrease in the number of oligodendroglia with age, while neurons, microglia, and astrocytes remain relatively stable across the lifespan (25). Microglia play a critical role in promoting oligodendrocyte precursor cell differentiation into mature myelinating oligodendrocytes (31) as this maturation is inhibited by the presence of myelin debris in the local neuro-environment (32)—debris that is normally cleared by microglia. Thus, microglia play a multifaceted role in promoting remyelination by oligodendroglia (33). With age, microglia become laden with lipofuscin, a remnant of phagocytosed material that accumulates and can interfere with the cell's ability to ingest more debris (34). With age, microglia are rendered less effective in clearing myelin and become chronically reactive as indicated by the increased density of amoeboid and hypertrophic microglia in the aged brain, which correlates with the severity of cognitive decline in aged monkeys (35). Along with failing to clear myelin debris, aged microglia are also characterized by an increase in the secretion of pro-inflammatory cytokines which are known to negatively impact cognition and increase production of reactive oxygen species (ROS) that can directly act to breakdown the lipids of the myelin sheath (36, 37). Together these data suggest that microglia, the main immune system cell of the brain, may contribute to age-related myelin pathology and white matter loss.

In addition to their likely role in exacerbating myelin damage, senescent microglia contribute to the more general phenomenon known as “inflammaging;” a term that has been used to describe the chronic increase in pro-inflammatory cytokines and chemokines in the aging CNS (38, 39). Systemic inflammation increases with age and is believed to be driven by dysregulation of the innate immune system, specifically by means of monocyte over-activation and secretion of pro-inflammatory molecules (40). Age-related neuroinflammation has been documented across species from rodents to humans and has been shown to be associated with normal cognitive impairment as well as age-related neurodegenerative diseases (41–43). In aging rodents, increased secretion of pro-inflammatory cytokines is accompanied by a decrease in cognitive performance, while those animals with more anti-inflammatory cytokines remain more cognitively spared (43). It has been demonstrated that pro-inflammatory cytokines underlie decreased secretion of neurotrophins and inhibit neurogenesis (44, 45). In our primate model of normal aging and white matter degeneration, we have shown that microglia exist in a chronic activation state and exhibit signs of increased phagocytic activity, states that correlate with severity of cognitive impairment (35). While changes to microglia, cells of the innate immune system, in brain aging have been characterized, less is known about how cells of the adaptive immune system, such as T cells, might be involved with CNS aging.

Conventional wisdom regarding immune privilege of the brain held that cross-talk between the CNS and the peripheral immune system was largely restricted by the blood brain barrier (BBB) (unless it was damaged by disease or trauma), preventing immune cells of the peripheral blood from entering the CNS. Reexamination of these issues has shown that CNS-antigen specific T lymphocytes reside at CNS border zones such as in the meninges and choroid plexus and exert a variety of effects on the brain including playing a critical role in normal cognitive function (46, 47). Thus, until recently, it has been thought that T cells of the peripheral immune system only cross the healthy BBB to survey the brain environment for infection, after which they exit back into the peripheral circulation *via* lymphatic drainage of the brain (48). With age, T cells at the choroid plexus undergo a shift from a more homeostatic profile to a more detrimental proinflammatory profile, which is negatively associated with cognition (49, 50). Moreover, a recent study also demonstrated T cell residence in the aging brain parenchyma where they have been shown to play a negative role in cognition by inhibiting hippocampal neurogenesis (51).

**Figure 1:**
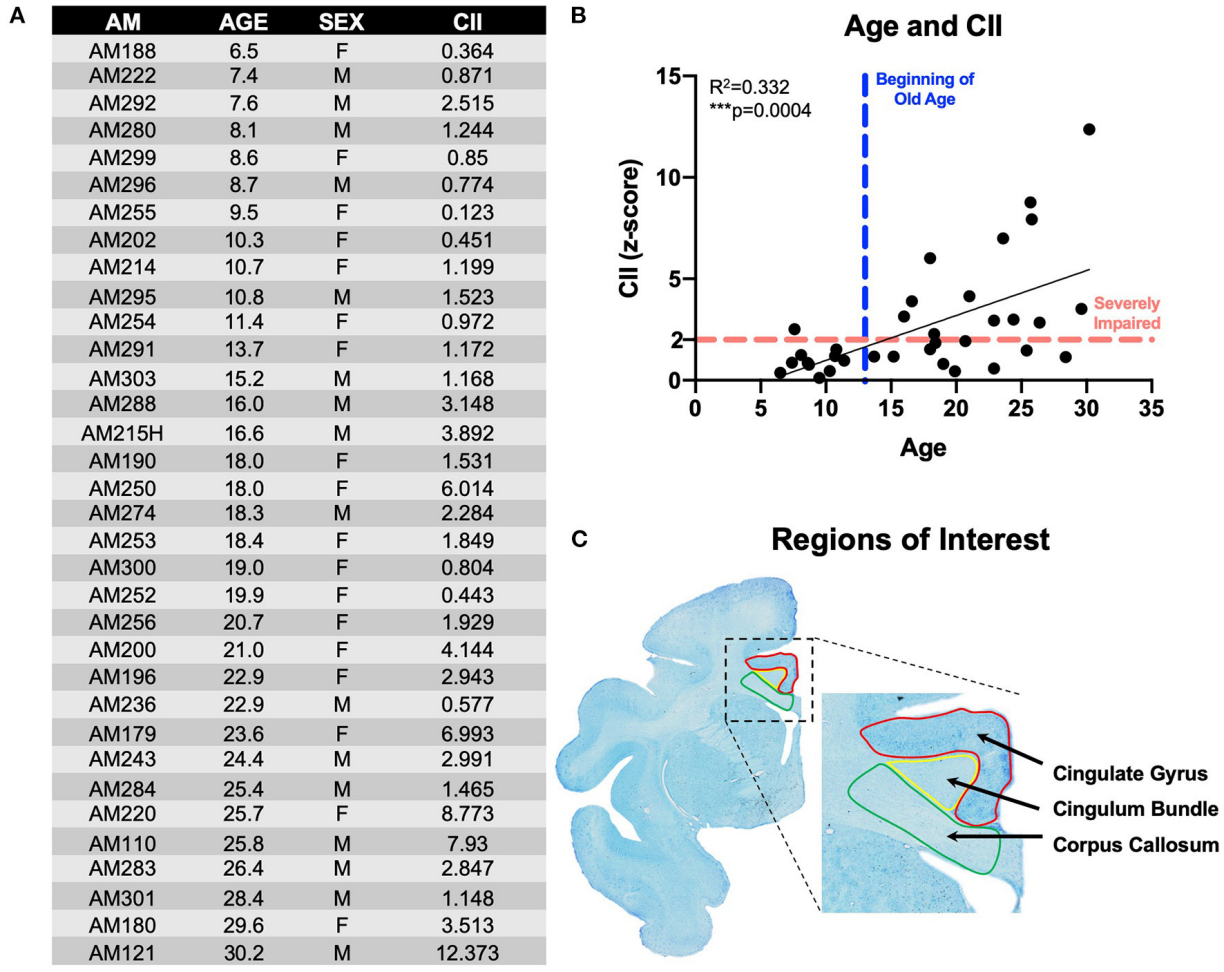
Subjects & experimental parameters: **(A)** Table listing the 34 rhesus monkeys used in these experiments with animal ID, age, sex, and cognitive impairment score; **(B)** Linear regression of animals' age and cognitive impairment index (CII) all 34 monkeys demonstrating age-related worsening of cognitive performance; **(C)** Thionin-stained section from animal AM301 showing the regions of interest used in these experiments with the cingulate gyrus (red), cingulum bundle (yellow), and corpus callosum (green). AM, aging monkey; CII, cognitive impairment index.

To determine if T cells are involved in white matter aging, we examined a cohort of 34 rhesus monkeys of different ages and both sexes that were cognitively characterized demonstrating varying degrees of age-related cognitive impairment (Figure 1B). Previous studies have shown that myelin sheath damage, particularly in the frontal white matter was the best predictor of age-related cognitive impairment (19, 21, 23, 52). Microglial activation and phagocytic dysfunction specifically in the aging white matter correlate with cognitive impairment severity and are hypothesized to play a central role in the age-related impairments in myelin sheath homeostasis (34, 35, 53–55). In the present study, we specifically asked whether myelin damage and chronic microglial activation in the frontal white matter of our aging monkeys are accompanied by a peripheral immune response of T cells. We demonstrate that not only do T cells surrounding blood vessels increase with aging, but T cells also infiltrate the white matter parenchyma where they correlate with the degree of microglial reactivity and cognitive impairment. Here, we present the foundation for examining T cells as a novel player in normal age-related cognitive decline.

## Methods

### Subjects

Male and female rhesus macaques aged 5–30 years old—equivalent to human ages 15–90 years old (56)—were carefully selected to exclude subjects with comorbid disease or experimental manipulations that would confound studies of the aging brain and behavior (Figure 1A). While in the study, subjects were maintained in the Animal Science Center on Boston University Medical Campus (BUMC), which is fully accredited by AAALAC and managed by a licensed veterinarian. All procedures conformed to the NIH Guide for the Care and Use of Laboratory Animals and were approved by the Institutional Animal Use and Care Committee (IACUC) of BUMC.

### Behavioral Testing and Cognitive Impairment Index

Monkeys received a behavioral test battery to assess learning, memory and executive functions. This battery consists of delayed non-match to sample (DNMS), delayed recognition span (DRST), both object and spatial modalities, delayed response, and conceptual set-shifting tasks. These tasks are briefly detailed below and more detail can be found in (14, 57, 58), as well as a summary of how our cognitive impairment index is calculated from a subset of these tasks (13). Testing was conducted 5 days a week and used food rewards. Water is available *ad libitum* and a daily ration of chow, fruits, and vegetables is given each day after testing is complete.

### Delayed Non-match to Sample (DNMS) Task

The DNMS is a benchmark task of learning and recognition memory which measures the monkey's ability to distinguish between a recently presented familiar object and a novel object following a delay period of 10 s. Once this is learned additional tests of recognition memory after done after delays of 2 min and then 10 min. Output of the acquisition phase, 2-min delay phase, and 10-min delay phase are used as measurements for this task of learning and recognition memory.

### Delayed Recognition Span Task (DRST) Object and Spatial

This tests monkeys' working memory capacity by requiring that the monkey to identify a new stimulus among an increasing array of serially presented, familiar stimuli first using spatial then non-spatial (objects) stimuli. Output is the span of correct responses across trials as a measurement of working memory.

### The Delayed Response (DR) Test

This tests monkeys' spatial working memory by assessing the monkey's ability to correctly identify, following various delays, the location of a food reward they previously saw hidden.

### Conceptual Set Shifting Task

This tests the monkeys' executive functioning by having the monkey learn rules not explicitly learned. Much like the Wisconsin Card Sorting Task, once the task is learned, the “rule” is switched and the monkey must shift to learn the new rule. Output is based on the errors the monkey makes and specifically the perseverative errors made after set shifting as a measure of executive function.

### Calculation of Cognitive Impairment Index (CII)

A principal components analysis revealed that scores on DNMS-acquisition, DNMS-2-min delays, and DRST-spatial were the best predictors of age-related cognitive impairment. From this subset of scores, a weighted average is computed and converted into a *z*-score that is normalized to the mean performance of a cohort of a reference group of 29 young, healthy, male monkeys. This constitutes a cognitive impairment index (CII) for each monkey as a representation of their overall learning and memory capacity. Thus, a CII (*z*-score) of <1.0 reflects no impairment, 1.0–2.0 reflects a mild impairment, while scores above 2.0 are considered severe cognitive impairment (13). The cognitive impairment distribution for the 34 rhesus monkeys examined in this study is presented in Figure 1B.

### Brain Tissue Harvest and Processing

Upon completion of behavioral testing, brains were harvested for tissue processing and molecular experiments. Detailed descriptions of tissue collection, storage, and preparations are in (59, 60). Briefly, monkeys were euthanized by exsanguination during a two-stage transcardial perfusion of the brain that begins with Krebs buffer at 4°C to clear vasculature and cool the brain to slow proteolysis while allowing fresh tissue dissection from one hemisphere for molecular experiments. Once fresh tissue collection is complete, perfusate is switched to 4% paraformaldehyde (37°C) to ensure full fixation of the intact hemisphere. The brain is then blocked *in situ* in the coronal stereotactic plane and stored overnight in 4% paraformaldehyde at 4°C. The fixed brain tissue (including an entirely intact hemisphere) are then removed from fixative, rinsed, cryoprotected in buffer with glycerol (61), flash frozen and stored at −80°C until cut into 10 interrupted series of 30 μm-thick sections so that sections in a series are spaced at 300 μm. Cut sections were stored in buffer with 15% glycerol at −80°C until removed as a group for batch processing, a process that does not affect immunohistochemistry (59).

### Immunohistochemistry

To identify CD3^+^ T cells, tissue sections from all relevant cases were selected to obtain 6–9 sections, each spaced 2,400 μm apart, per animal to analyze the cingulum bundle, anterior body and rostrum of the corpus callosum, and cingulate gyrus (Figure 1C). These regions were chosen for their known age-related myelin damage in humans and our monkey model of normal aging (10, 19). The gray matter of the cingulate gyrus was chosen as a neighboring control region unaffected by normal white matter aging. Tissue from all cases was removed from the freezer, thawed at room temperature and batch processed in the same reagents at the same time to eliminate processing variability. Sections were rinsed in 0.05 M Tris-buffered saline (TBS) at pH 7.6 to remove glycerol. To break cross-links formed during fixation, antigen retrieval was then performed by incubating tissue in Tris-EDTA buffer at pH 9 in a microwave tissue processor (PELCO Biowave, Ted Pella, Inc. Redding, CA) for 10 min at 40°C and 550 W power followed by incubation in the same buffer at room temperature for 1 h. Sections were then washed with TBS (3 × 5 min) and blocked for 1 h in SuperBlock (Life Technologies, Grand Island, NY) at room temperature followed by incubation in CD3 primary antibody (BioRad, monoclonal rat anti-CD3, clone CD3-12, MCA1477) diluted to 1:500 in TBS with 0.5% SuperBlock and 0.3% Triton X-100 (Sigma) for 24 h at room temperature on a rocker. Control sections were incubated in the same solution lacking the primary antibody. After incubation, tissue was washed in TBS (3 × 5 min) and quenched in 3% H_2_O_2_ for 30 min at room temperature to inactivate endogenous peroxidases. Tissue was washed again in TBS (3 × 5 min) and incubated in secondary antibody solution in TBS containing 0.5% Superblock, 0.3% Triton X, and a 1:600 concentration of biotinylated goat anti-rat secondary (Vector Laboratories, Burlingame, CA) for 1 h at room temperature. Tissue was washed in TBS (3 × 5 min) and then incubated in avidin-biotin complex using Vectastain ABC kit (Vector Laboratories, Burlingame, CA) for 1 h at room temperature. Following incubation, tissue was washed in TBS (3 × 5 min) and then incubated in a chromogen solution containing 0.5 mM 3-3-diaminobenzadine (Sigma-Aldrich, St. Louis, MO) and 0.03% hydrogen peroxide in TBS for 10 min. Tissue sections then went through a final washing in TBS (3 × 5 min) and were stored at 4°C until mounted on gelatin coated slides and air-dried for 48 h before being dehydrated through alcohols and cleared in xylenes (2 × 20 min). Slides were coverslipped with Permount (Fisher Scientific, Waltham, MA), arranged anatomically and blinded for quantification.

Fibrinogen staining was performed on 3 tissue sections spaced 7,200 μm apart per subject from a subset of the cohort to analyze the cingulum bundle, anterior body, and rostrum of the corpus callosum, and cingulate gyrus (Figure 1C). The same IHC protocol as described above was used, with primary antibody (DAKO, polyclonal rabbit anti-fibrinogen, A0080) at a concentration of 1:5,000 and secondary antibody (biotinylated goat anti-rabbit, Vector Laboratories, Burlingame, CA) at a concentration of 1:1,000.

To identify LN3^+^ microglia, tissue sections were selected from a subset of the same subjects that were over the age of 20 years old to obtain 6–9 sections, each spaced 2,400 μm apart, per animal to analyze the cingulum bundle, anterior body and rostrum of the corpus callosum, and cingulate gyrus (Figure 1C). For IHC, sections were removed from the freezer and thawed as described above and processed through the same steps as the CD3 series except without the initial antigen retrieval step. Primary LN3 antibody (Thermo Fisher, monoclonal mouse, anti-LN3, clone LN3, MA1-35420) was used at a concentration of 1:100 and secondary was used at a concentration of 1:600 (biotinylated anti-mouse Vector Laboratories, Burlingame, CA). Color precipitation was performed with the same chromogen solution described above with the addition of 0.1 M nickel sulfate.

Multi-label immunofluorescence was performed as described above but with a 2-h blocking step, overnight incubation in primary antibodies to CD3 (1:500, BioRad, monoclonal rat anti-CD3, clone CD3-12, MCA1477), collagen IV (1:500, Sigma, monoclonal mouse anti-collagen IV, clone COL-94, C1926), and GFAP (1:1,000, Dako, polyclonal rabbit anti-GFAP, Z0334), followed by a 2-h incubation in appropriate fluorescent labeled secondary antibodies. Tissue was slide-mounted and imaged using a Zeiss LSM 710 NLO confocal microscope at high magnification (40X oil objective).

### T Cell and Microglia Quantification

**Figure 2:**
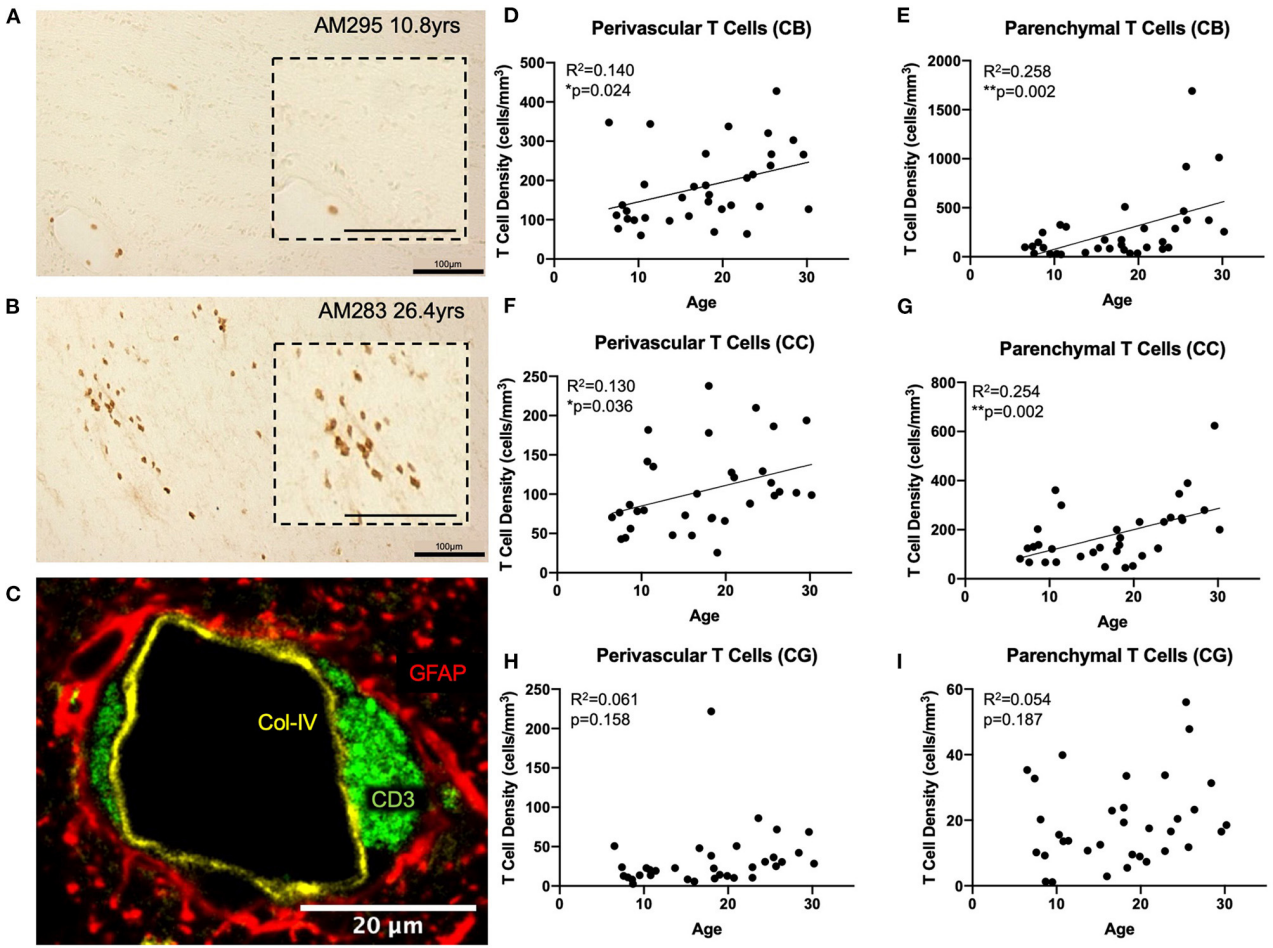
T cells infiltrate the white matter parenchyma in aging: **(A)** CD3^+^ IHC staining (brown) in the white matter of a young and an old **(B)** monkey illustrating the increase in density of parenchymal T cells in the aged brain. Insets show an enlarged view; **(C)** Fluorescent multi-labeling to show that perivascular T cells (CD3^+^ in green) reside in the glymphatic space between the basement membrane (collagen IV in yellow) and the astrocytic endfeet (GFAP in red). **(D,E)** both perivascular and parenchymal T cells significantly increase in the cingulum bundle with age; **(F,G)** Perivascular and parenchymal T cells significantly increase in the corpus callosum with age; **(H,I)** T cells do not increase in the gray matter of the cingulate gyrus with age. CB, cingulum bundle; CC, corpus callosum; CG, cingulate gyrus. *N* = 34.

Unbiased stereology was performed on a Nikon E600 light microscope equipped with a Q-Imaging digital camera, a motorized stage and StereoInvestigator software (MBF Bioscience, Williston, VT). The regions of interest (cingulum bundle, corpus callosum, and cingulate gyrus) were identified and demarcated (Figure 1C) at low magnification (1X objective). The optical fractionator method as described previously (62) and applied to monkey tissue (60) was used to quantify CD3 and LN3 positive cells within each of these ROIs at high magnification (20X objective). Briefly, a sampling grid was placed with a randomized starting location over each outlined ROI. Within each sampling grid square, a counting frame was placed with a dissector top guard volume extending to 2 μm below the tissue section surface. Cells not intersecting exclusion planes were counted. Grid size and counting percentage was optimized to minimize the coefficient of error (CE), which is calculated as previously reported (63). CE values were ≤ 0.10 in all ROIs except the cingulate gyrus gray matter and for other ROIs in young animals. In those cases, CE values below 0.1 were unattainable due to low total number of countable T cells. The Cavalieri estimator was used to calculate the volume of each ROI. As they were counted, CD3^+^ T cells were classified as “parenchymal” if they did not border a blood vessel. “Perivascular” T cells were classified as those surrounding a vessel (Figures 2A–C). LN3^+^ microglia were identified as ramified, hypertrophic, or amoeboid based on their morphology, as previously described (64).

### Fibrinogen Quantification

The same microscope setup described above was used to first outline the regions of interest (cingulum bundle, corpus callosum, and cingulate gyrus) at low magnification (1X objective). A grid was placed over each ROI so that systematic random sampling could be performed to acquire images for fibrinogen stain analysis. An image was collected at high magnification (20X objective lens) at every 6th grid site for the cingulum bundle, every 8th for the corpus callosum, and every 15th for the cingulate gyrus. Image J software (NIH) was used to convert images to 32-bit and auto-thresholded (“Default” setting) to then calculate percent area stained. Measurements within the same region of the same animal were averaged to obtain a single value representing the average percent area stained per region per subject.

### Statistics

All variables including ages are treated as continuous numerical values. All regression analyses were performed on independent measures using Prism with an alpha significance value of 0.05.

## Results

### T Cells Infiltrate the White Matter Parenchyma of the Aged Brain

We used anti-CD3 to label all T cell subtypes in the CNS. Initial observation of tissue sections stained with CD3 antibody revealed the expected presence of CD3 positive T cells in the choroid plexus, ependyma lining the ventricles, and meninges as previously reported (49, 65, 66). However, it was also noted that T cells were present surrounding the penetrating vasculature as well as within the white matter parenchyma, primarily in the aged brain (Figures 2A,B). We designated these populations as perivascular and parenchymal, respectively.

To confirm the spatial relationship of perivascular T cells to the vasculature, we performed multi-label immunofluorescence using antibodies against CD3 (T cells), collagen IV (basement membrane of endothelium), and GFAP (glial fibrillary acidic protein—to label astrocytic endfeet around blood vessels) and found that these perivascular T cells are located in the “glymphatic space” (67), proximal to the astrocyte investment but outside the basement membrane so that they are restricted from direct contact with the brain parenchyma (Figure 2C). In contrast, parenchymal T cells were not associated with any vascular elements but instead were located within the brain tissue. Quantification distinguished these as unique due to their localization within the white matter.

Tissue stained for CD3 from 34 rhesus monkeys (16 male and 18 female) ages 5–30 years old (see Figure 1A) was analyzed to quantify parenchymal and perivascular T cell density in three regions of interest—the white matter of the cingulum bundle and corpus callosum, and the gray matter of the cingulate gyrus. As shown in Figures 2D–G, perivascular T cell density significantly increases with age in the cingulum bundle [*F*_(1, 32)_ = 5.203, *R*^2^ = 0.1399, *p* ≤ 0.05] and the corpus callosum [*F*_(1, 32)_ = 4.79, *R*^2^ = 0.1302, *p* ≤ 0.05]. Parenchymal T cell density also significantly increases with age in the cingulum bundle [*F*_(1, 32)_ = 11.10, *R*^2^ = 0.2575, *p* ≤ 0.05] and corpus callosum [*F*_(1, 32)_ = 10.88, *R*^2^ = 0.2537, *p* ≤ 0.05]. In the gray matter of the adjacent cingulate gyrus, T cells were rare in both the perivascular and parenchymal compartments and neither showed any significant difference with age [perivascular *F*_(1, 32)_ = 2.092, *R*^2^ = 0.06136, *p* = 0.1578 and parenchymal *F*_(1, 32)_ = 1.815, *R*^2^ = 0.05368, *p* = 0.1874] (Figures 2H,I).

### Percentage of CNS T Cells in the Parenchyma Increases With Age

**Figure 3:**
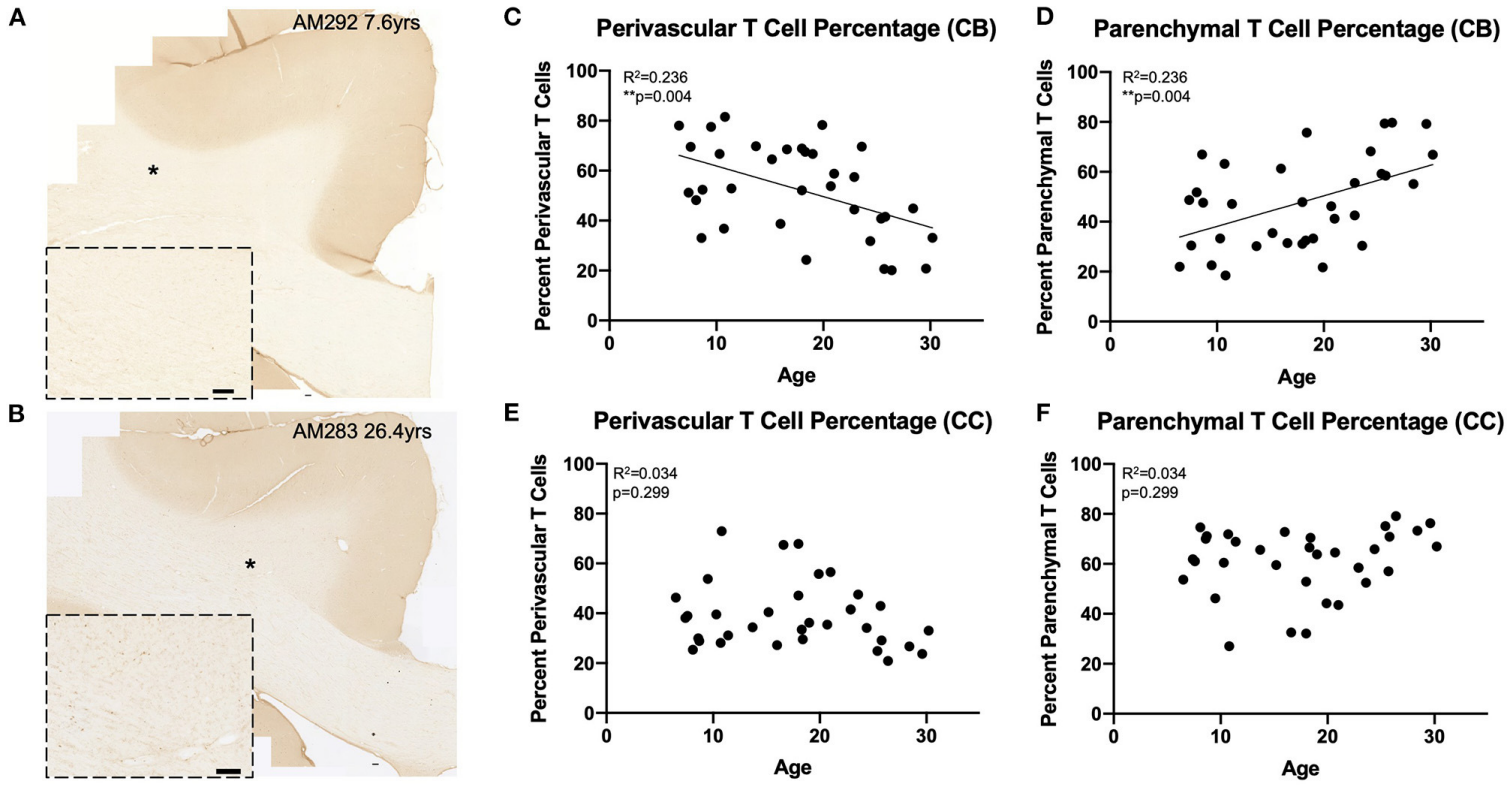
Greater proportion of T cells in the white matter parenchyma with age: **(A,B)** Representative region of interest images for CD3^+^ stained T cells in a young **(A)** and old **(B)** monkey. Inset shows detail, location of inset marked with * in large image. Scale bars are 100 μm. **(C,D)** The percentage of perivascular T cells decreases while the percentage in the parenchyma increases with age in the cingulum bundle; **(E,F)** The percentage of perivascular T cells as well as those in the parenchyma does not change with age in the corpus callosum. CB, cingulum bundle; CC, corpus callosum. *N* = 34.

The perivascular T cells observed here could represent cells that are either entering or exiting the parenchyma (68), or behaving as resident border-zone regulators, which exert a variety of functions *via* cytokine signaling (46, 66). Because of this, we compared the percentages of T cells in the parenchyma (parenchymal/(parenchymal + perivascular)) to the percentage of T cells in the perivascular space across age in the cingulum bundle and corpus callosum (Figures 3A,B). As shown in Figures 3C,D, we found that in the cingulum bundle the percentage of T cells in the perivascular space was reduced while there was a complementary increase in the percentage of T cell within the parenchyma [*F*_(1, 32)_ = 9.86, *R*^2^ = 0.236, *p* ≤ 0.05]. This suggests an enhanced parenchymal accumulation, which could be due to increased entry, decreased egress, or a combination of both. In contrast, as shown in Figures 3E,F, in the corpus callosum there was no significant shift with age in the percentage of T cells in the perivascular space compared to the parenchyma [*F*_(1, 32)_ = 1.11, *R*^2^ = 0.0336, *p* = 0.2991].

### T Cell Infiltration Into the Cingulum Bundle Correlates With Microglial Reactivity

**Figure 4:**
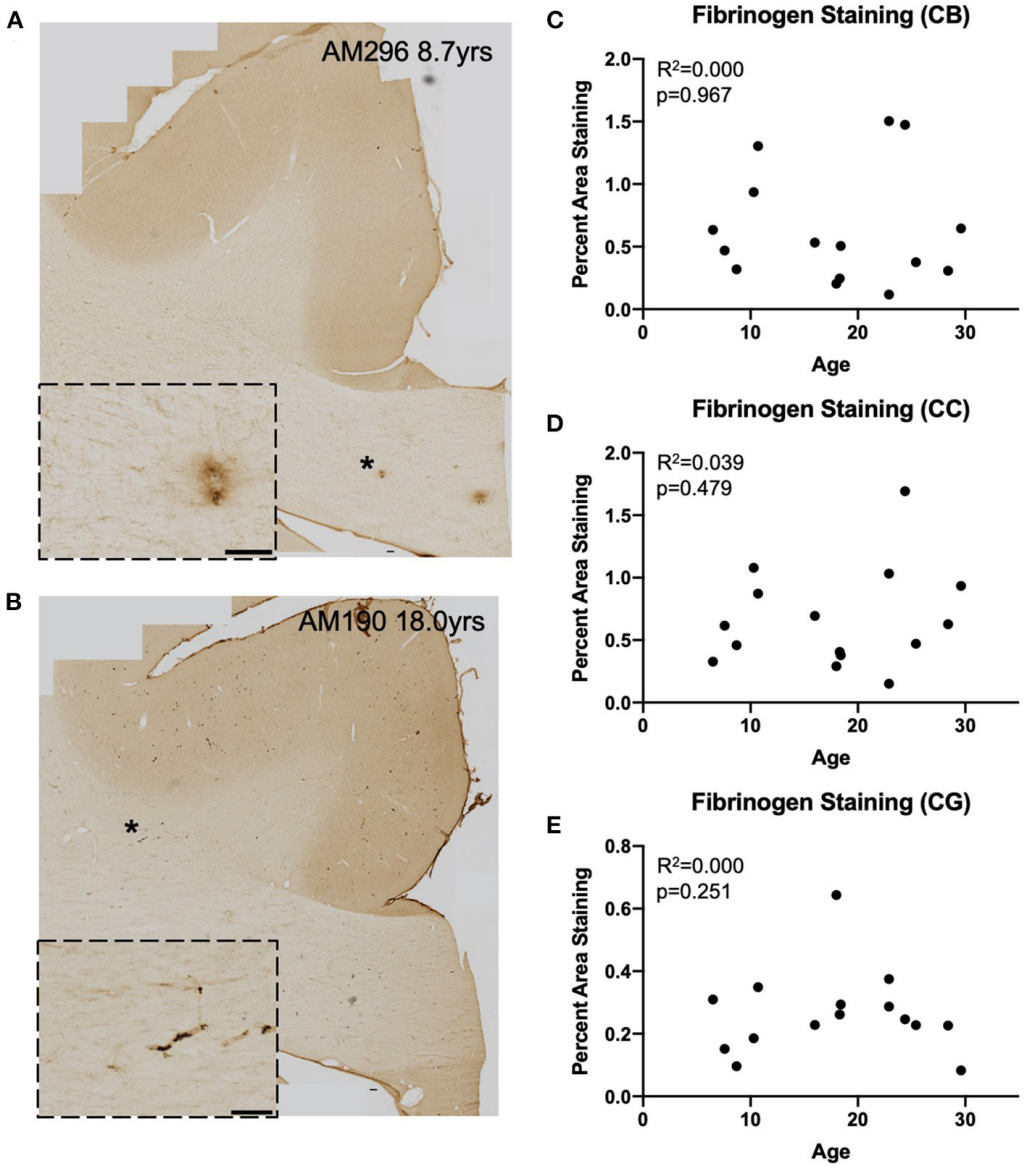
Fibrinogen staining with age: IHC for fibrinogen was used to assess possible leakage from the vasculature in a representative subset (*n* = 15) of monkeys **(A,B)** Representative images to exhibit the variability in fibrinogen staining between subjects. Insets show detail of vascular and glial staining that were quantified as positive signal. Location of inset marked with * in large image. Scale bars are 100 μm. **(C–E)** There is an absence of significant age-related fibrinogen staining as percent are in the cingulum bundle **(C)**, the corpus callosum **(D)**, and the gray matter of the cingulate gyrus **(E)**. CB, cingulum bundle; CC, corpus callosum; CG, cingulate gyrus.

**Figure 5:**
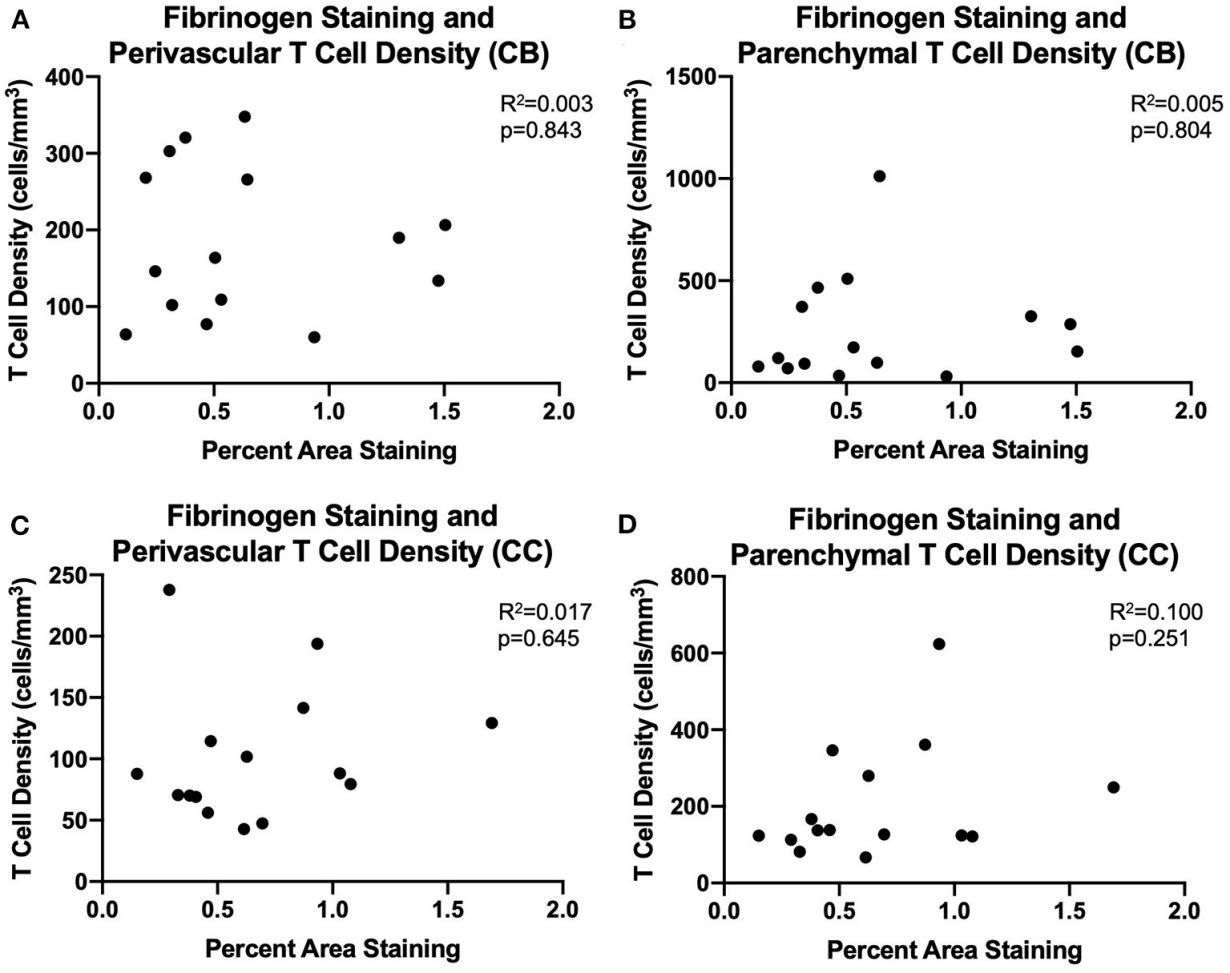
BBB leakiness does not account for T cell infiltration into the white matter: **(A–D)** Fibrinogen presence measured by percent area staining did not correlate with perivascular or parenchymal T cell density in the cingulum bundle or the corpus callosum. CB, cingulum bundle; CC, corpus callosum. *N* = 15.

We assessed whether T cells were leaking into the brain parenchyma through a damaged blood brain barrier by performing immunohistochemistry to quantify fibrinogen in the brain parenchyma. Fibrinogen is a large (340 kDa) coagulation protein found in blood serum that should not be present in the brain if BBB is intact, allowing immunohistochemical staining and quantification of fibrinogen in the brain to be used to assess BBB leakiness for molecules smaller than 340 kDa (69). Fibrinogen staining in a subset of 15 monkeys (8 male and 7 female) aged 6.5–29.6 years old was quantified as percent area stained in the cingulum bundle, corpus callosum, and cingulate gyrus. In the gray matter of the cingulate gyrus staining was almost exclusively associated with the vasculature. White matter staining varied a bit between subjects and ranged from predominantly vascular associated to some glial staining of fibrinogen because it binds to CD11b receptors (70–72) (Figures 4A,B). As shown in Figures 4C–E, the percent area of fibrinogen staining in the brain parenchyma was minimal, ranging from 0.05 up to 1.7% across all ROIs and cases, confirming previous reports in a study of AD brains of fibrinogen staining in age-matched healthy controls reflecting BBB integrity (73). Importantly, staining did not change with age in the cingulum bundle [*F*_(1, 13)_ = 0.00178, *R*^2^ = 0.000137, *p* = 0.9670], corpus callosum [*F*_(1, 13)_ = 0.531, *R*^2^ = 0.0392, *p* = 0.4791], or cingulate gyrus [*F*_(1, 13)_ = 0.000166, *R*^2^ = 1.28e-005, *p* = 0.9899]. Since T cells are much larger (5–7 μm) than fibrinogen (340 kDa), it was unlikely that age-related T cell infiltration was due to breakdown of the BBB. Nevertheless, as a test of whether T cells might be associated with this low level of fibrinogen, we examined the relationship of fibrinogen levels to perivascular and parenchymal T cell density. As shown in Figures 5A–D, there was no correlation between T cell infiltration and the amount of fibrinogen in the cingulum bundle [perivascular *F*_(1, 13)_ = 0.0409, *R*^2^ = 0.00313, *p* = 0.8429 parenchymal *F*_(1, 13)_ = 0.0643, *R*^2^ = 0.00492, *p* = 0.8038] or the corpus callosum [perivascular *F*_(1, 13)_ = 0.222, *R*^2^ = 0.0168, *p* = 0.6454, parenchymal *F*_(1, 13)_ = 1.44, *R*^2^ = 0.0999, *p* = 0.2512], suggesting that T cell infiltration does not occur across a leaky BBB in these monkeys.

**Figure 6:**
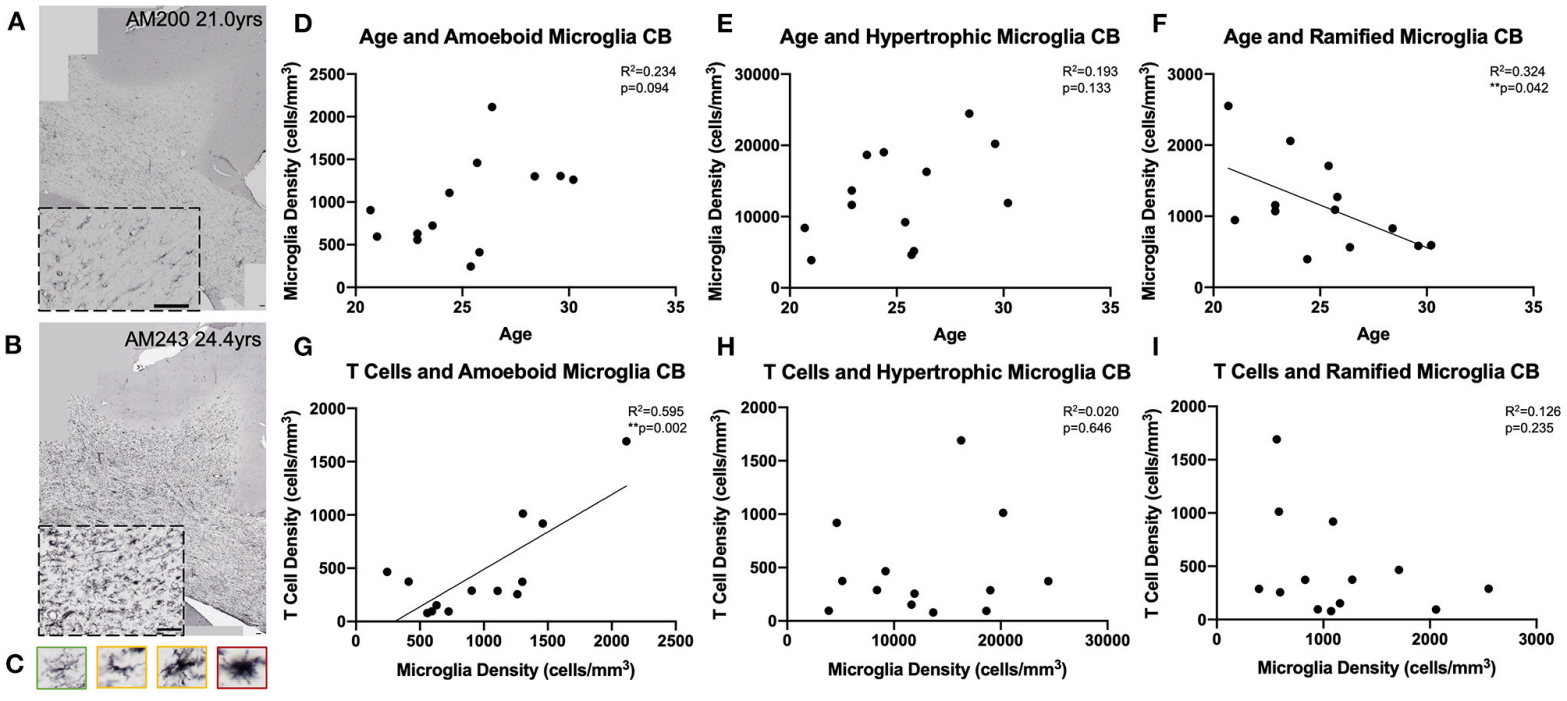
Microglial reactivity correlates with T cell infiltration in the cingulum bundle: **(A,B)** Representative region of interest images depicting lower **(A)** and higher **(B)** levels of microglial reactivity with LN3 staining. **(C)** Microglial reactivity in the cingulum bundle was quantified by counting the density of LN3^+^ stained cells by morphology, grouping them into ramified (green outline), hypertrophic (yellow outline), and amoeboid (red outline). The density of amoeboid **(D)** and hypertrophic **(E)** microglia did not change with age, however there was a significant decline in the density of ramified microglia with age **(F)**. The density of amoeboid microglia correlated with the density of T cells that infiltrated the cingulum bundle parenchyma **(G)**. T Cell density did not correlated with hypertrophic **(H)** or ramified **(I)** microglia density. CB, cingulum bundle. *N* = 13.

We next examined whether T cell entry into the brain may be in response to increases in microglial reactivity. To assess whether the observed age-related increase in microglial reactivity in the oldest subjects of this cohort (≥20 years old, *n* = 13) might be related to infiltrating T cells, we stained tissue of the same section spacing as CD3 with the LN3 antibody (anti-MHC-II receptor) (Figures 6A,B). We were specifically curious about microglia that are involved in antigen presentation *via* MHC-II receptors, which have been suggested to stimulate and activate CNS T cells (74). As described by Karperien et al. (64) we classified LN3 positive microglial subtypes by their morphology as ramified, hypertrophic, or amoeboid microglia reflecting the least to most inflammatory/phagocytic phenotypes (Figure 6C). Quantifying microglia by this classification scheme we found in the cingulum bundle age-related increases in density that approached significance for both amoeboid [*F*_(1, 11)_ = 3.36, *R*^2^ = 0.234, *p* = 0.0941] and hypertrophic [*F*_(1, 11)_ = 2.63, *R*^2^ = 0.193, *p* = 0.1330] microglia (Figures 6D,E). There was a significant decrease in the density of ramified microglia with age [*F*_(1, 11)_ = 5.27, *R*^2^ = 0.324, *p* ≤ 0.05] (Figure 6F). These results are aligned with previous findings in our model that LN3^+^ amoeboid microglia increase in the cingulum bundle with age (35). We then examined whether this increased microglial reactivity is related to T cell infiltration and found that an increase in amoeboid microglial density significantly correlated with higher density of parenchymal T cells [*F*_(1, 11)_ = 16.2, *R*^2^ = 0.595, *p* ≤ 0.05] (Figure 6G). There was no relationship between hypertrophic [*F*_(1, 11)_ = 0.224, *R*^2^ = 0.0199, *p* = 0.6455) or ramified [*F*_(1, 11)_ = 1.58, *R*^2^ = 0.126, *p* = 0.2346) microglia density and T cell density (Figures 6H,I). These data suggest that T cell infiltration is associated with increased neuroinflammation as quantified by density of pro-inflammatory microglia.

### Higher Percentage of Parenchymal T Cells Correlates With Cognitive Impairment

**Figure 7:**
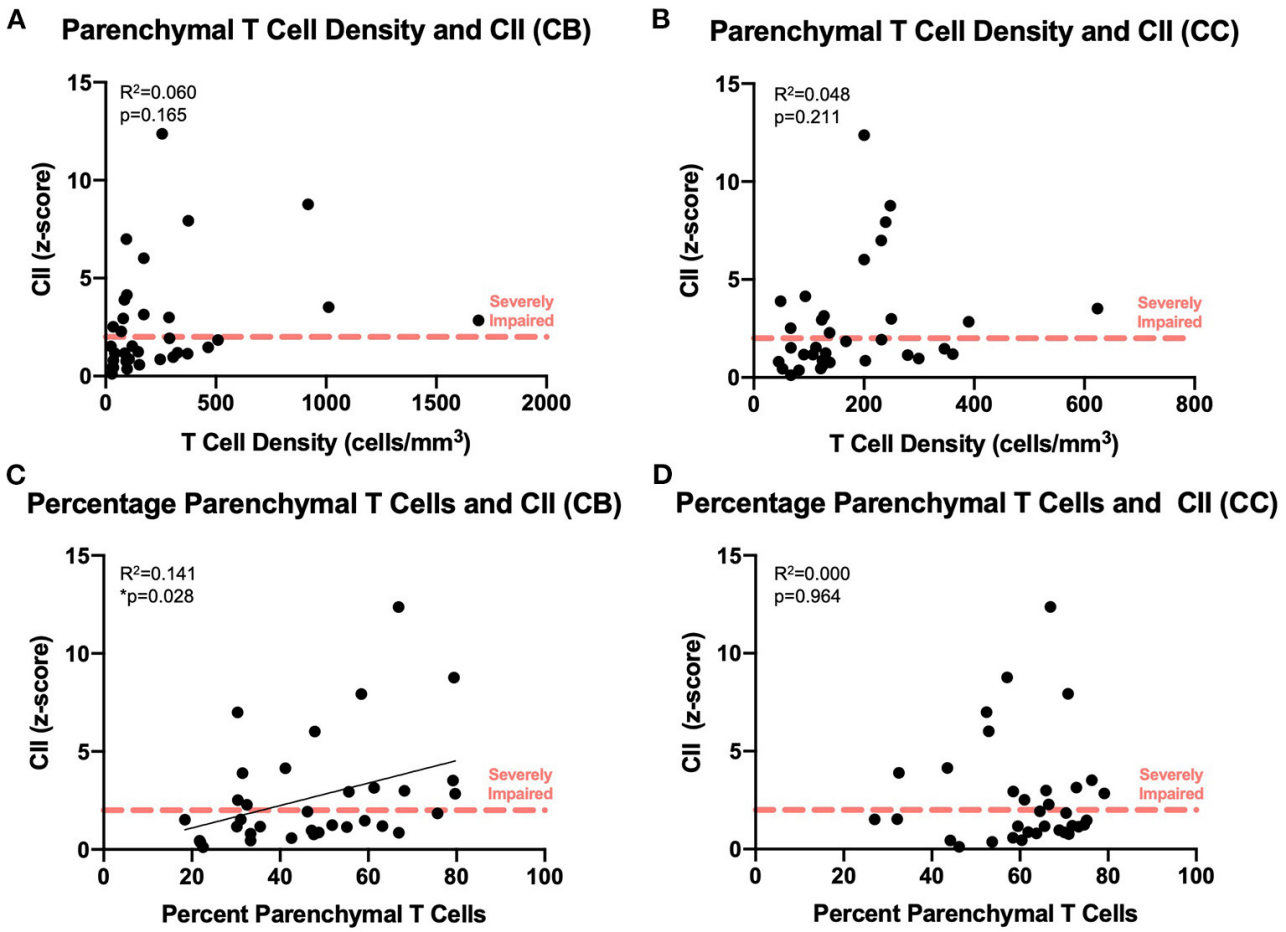
Increased percentage of parenchymal T cells in the cingulum bundle correlates with worsening cognitive performance: The density of parenchymal T cells does not correlate with cognitive impairment in the cingulum bundle **(A)** or the corpus callosum **(B)**; the percentage of T cells in the parenchyma correlates with the cognitive impairment index in the cingulum bundle **(C)** but not in the corpus callosum **(D)**. CB, cingulum bundle; CC, corpus callosum; CII, cognitive impairment index. *N* = 34.

We compared our findings of T cell density to the cognitive scores established for each subject as previously described (Figure 1B). As shown in Figures 7A,B, there was no significant relationship between absolute density of parenchymal T cells and the CII in the cingulum bundle [*F*_(1, 32)_ = 2.02, *R*^2^ = 0.0595, *p* = 0.1646] or in the corpus callosum [*F*_(1, 32)_ = 1.63, *R*^2^ = 0.0484, *p* = 0.2113]. In contrast, when T cell accumulation in the parenchyma was assessed by percentage parenchymal T cells as described above—parenchymal/(parenchymal + perivascular)—there was a significant relationship between the percentage of T cells in the parenchyma of the cingulum bundle and cognitive impairment (CII) [*F*_(1, 32)_ = 4.86, *R*^2^ = 0.132, *p* ≤ 0.05] but not in the corpus callosum [*F*_(1, 32)_ = 0.0132, *R*^2^ = 0.000413, *p* = 0.9092] (Figures 7C,D). This is in agreement with previous studies showing that neither white matter damage nor neuroinflammation in the corpus callosum are predictive of age-related cognitive decline in the rhesus monkey even when these changes in the cingulum bundle are (19, 35).

## Discussion

### Summary of Results

Primate and human aging are characterized by varying degrees of cognitive decline caused by an accumulation of damaged myelin sheaths rather than neuronal degeneration like that observed in AD (3–7, 19, 75). Myelin sheath pathology has been previously characterized in the frontal white matter of our primate model of normal cognitive aging (19, 23, 76, 77). Closer examination of these regions revealed a role of activated, dystrophic microglia that correlated with the severity of cognitive impairment (35). Here we set out to examine whether T cells may be responding to increased neuroinflammation and myelin damage in brain regions known to be vulnerable to age-related myelin pathology in the normal aging rhesus monkey. The main findings of this study are: (1) In normal aging, T cells that are normally excluded from the brain in healthy young adults, increase in the perivascular space surrounding cortical blood vessels and infiltrate into the parenchyma of the white matter but not the gray matter. (2) In the white matter of the cingulum bundle but not the adjacent corpus callosum, the percentage of CNS T cells that infiltrate and accumulate in the white matter parenchyma increases with age compared to those in the perivascular space. (3) T cells do not passively “leak” into the brain through a damaged BBB but instead infiltrate in a manner that correlates with proinflammatory microglia classified as amoeboid (phagocytic), suggesting an active process. (4) Most importantly, the percentage of parenchymal T cells in the cingulum bundle correlated with age-related impairments in cognitive function. These data suggest that T cells in the white matter may directly contribute to age-related white matter damage and cognitive decline in the healthy, aged brain. Hence, T cells infiltrating the CNS parenchyma may contribute to white matter damage making them a target for future therapeutic interventions aimed at ameliorating cognitive aging.

### Cognitive Impairment and Neuroinflammation in Normal Aging

Cognitive decline in normal, non-neurodegenerative aging, occurs in monkeys (23) and humans (3, 7). Careful studies that exclude early stage Alzheimer's cases show that these age-related cognitive impairments are not the result of the degeneration or loss of cortical neurons but likely result, at least in part, from an age-related accumulation of myelin sheath damage followed by subsequent demyelination and axon loss producing a cortical “disconnection” (19, 21, 24). The processes leading to myelin damage and the failure of myelin sheath repair in normal aging are not clearly understood, but it has been suggested that oxidative damage and inflammation contribute to damage of myelin lipids and oligodendrocyte senescence (78–80). In normal aging monkeys, an age-related increase myelin debris could overburden the phagocytic capacity of microglia leading to phagocytic inefficiency and accumulation of interstitial myelin debris. This in turn can impair maturation of oligodendrocyte precursor cells into mature, myelinating oligodendrocytes, reducing their ability to repair age-related myelin sheath damage (26–28, 31, 32, 34, 81). Moreover, there is a plethora of data demonstrating a generalized, age-related increase in markers of inflammation both in the periphery and within the CNS, often referred to as “inflammaging” (82, 83).

Neuroinflammation is known to contribute to many age-related neurodegenerative diseases as well as normal age-related white matter damage (34, 82). The density of LN3 positive activated and Gal-3 positive phagocytic microglia increase specifically in frontal white matter tracts with age and correlate with cognitive impairment (19, 35). Further, in the aging brain, microglia appear to be chronically pro-inflammatory, with increased expression of IL-1β, IL-6, and TNF-α and decreased expression of anti-inflammatory cytokines such as IL-10 (36). In the periphery, secretion of pro-inflammatory cytokines by macrophages can lead to recruitment and activation of T cells (84). Here, we have confirmed an age-related increase in the pro-inflammatory phenotype of microglia within the white matter and further demonstrate a positive relationship between age-related increased inflammatory microglia and increased density of T cells within the white matter parenchyma.

In addition to clearance by microglial phagocytosis, myelin debris has been found to be cleared from the brain by the CSF where it may drain through the lymphatics into the deep cervical lymph nodes (67, 85, 86). Once myelin debris enters the peripheral circulation, it can interact with antigen presenting cells (APCs) in the lymph nodes. This process has been observed in many demyelinating diseases and corresponding animal models (87). Of note, myelin-reactive T cells have been observed in the periphery of healthy individuals lacking gross myelin pathology (85). With age, myelin basic protein increases in the CSF of our aged monkeys (unpublished data), which would enable increased antigen exposure to the adaptive immune system and could explain why T cells may become myelin reactive and traffic to specific white matter regions in aging brain, though this remains to be confirmed in our aging monkeys.

### T Cell Trafficking Into the CNS

In Alzheimer's and other neurodegenerative diseases, there is a breakdown of blood brain barrier integrity, which may, at least in part, account for the T cell infiltration reported in human brain (73, 88–90). Here, we show that in the normal aging monkey brain, the minimal fibrinogen extravasation that we do observe is not associated with T cells. Hence, T cell infiltration into the brain in normal aging seems likely to be an active and regulated process and not a result of leakage through a damaged BBB.

The trafficking of T cells out of the vasculature and into inflamed tissues in the periphery relies on a process called “diapedesis,” which involves stepwise expression of receptors and ligands on endothelial cells (e.g., E- and P-selectins) and T cells (e.g., LFA-1) to facilitate slowing, rolling, adhesion and migration of T cells across the endothelium. Slowing and rolling are mediated by endothelial expression of selectins (E- and P-selectins), followed by their expression of integrins (ICAM and VCAM) (87, 91). Mechanisms of T cell entry into the CNS parenchyma are under debate, as it does not appear to follow the same sequence of steps observed in peripheral tissues and thus could serve to protect the brain's immune privilege status (68). One example of how the CNS protects its immune privilege status, is that astrocytes secrete factors that down-regulate the expression of E- and P-selectin on the BBB endothelium (92). Interestingly, in murine models of MS (i.e., EAE), it has been shown that T cell entry into the CNS is not dependent on E- and P-selectins as it is in humans but instead appears to rely exclusively on ICAM and VCAM expression on endothelial cells (92). Conversely, in the non-inflamed CNS, entry of T cells across the murine BBB seems to depend on E- and P-selectins and *not* ICAM or VCAM (93). As such, these differences among species has made it difficult to study concretely how T cells enter the CNS in health and disease.

For the non-inflamed CNS, the majority of studies agree that T cells can cross into the CSF across an intact BBB *via* the choroid plexus and/or the leptomeningeal blood vessels for surveillance (66, 87, 94). Yet, other studies have suggested migration can occur across the penetrating parenchymal vessels (95). To complicate the story further, studies have shown that E- and P-selectins are not expressed by the endothelium of the penetrating vessels in the human brain, rather this is exclusive to the endothelium of the meningeal blood vessels and those of the choroid plexus (96). Consequently, it is not possible to draw firm conclusions as to the directionality (entry or exit) of the perivascular T cells surrounding the penetrating parenchymal vessels in the aging monkey brain. However, we have shown an increase in the percentage of T cells within the parenchyma, which could suggest either increased entry, decreased exit, or a combination of both. Nevertheless, since this buildup of parenchymal T cells correlates with worsening cognitive performance, it is essential to obtain a greater understanding of trafficking mechanisms as this may provide avenues to reduce the density of CNS parenchymal T cells and prove beneficial to age-related cognitive decline.

### T Cells in the Diseased CNS

T cells residing at the choroid plexus are able to surveil the CSF and can cross the BBB upon antigen recognition in cases of injury or disease (66, 97). Following traumatic brain injury, microglia recruit first the innate immune system followed by cells of the adaptive immune system, including T cells, to the affected area where they participate in the local CNS immune response aimed at neurorestoration (98). Using an optic nerve crush injury model in rodents, researchers demonstrated that CNS-specific T cells (e.g., myelin recognizing) reduced neuronal loss (99). CNS-antigen recognizing T cells have often been considered largely detrimental due to their role in autoimmune diseases like MS (100). However, a more beneficial role of CNS auto-reactive cells has been termed “protective autoimmunity,” which protects neurons from secondary degeneration known to occur after initial injury (101). This protective role depends on a population of CNS antigen-specific memory T cells that reside in the meninges and choroid plexus and are primed to respond to damage following injury and promote beneficial neuroinflammation and lymphocyte recruitment for neuroprotection and repair (66, 88).

In Alzheimer's Disease, neuroinflammatory processes have been examined predominantly by looking at reactive astrocytes and microglia (102–104). Though early reports suggested the presence of T lymphocytes in diseased patient brains (105, 106), their role in AD has only been recently explored. It has been shown that AD plaques are surrounded by activated microglia that are likely interacting with CD8^+^ lymphocytes and might play a role in plaque pathology (107). Further, these AD-associated CD8^+^ T cells may contribute to synaptic plasticity dysfunction and preventing their infiltration results in beneficial restoration of neural plasticity gene expression programs (108). Characterization of T cells circulating in the CSF of patients with AD has revealed clonal expansion of CD8^+^ effector T cells with potential implications for neurodegeneration *via* their cytotoxic effector functions (109). Our data confirm these findings of age-related infiltrating T cells but we further characterize their infiltration specifically into white matter regions with known myelin pathology and correlate this influx of T cells with normal cognitive decline.

Much of our understanding of T cells' interactions with myelin comes from studies in human multiple sclerosis and corresponding animal models. These studies have shown that exposure of myelin epitopes to peripheral T cells may lead to auto-attack and demyelination as T cells initially sensitized in the periphery become reactivated upon entry into the CNS (110–112). Studies of MS therapeutics aimed at preventing T cell entry into the CNS uncovered a previously under-appreciated role of T cell surveillance for viruses and showed that blockade of T cells was potentially lethal (113). In the case of viral infection, such as JC virus, T cells specific to the virus are able to detect the presence of infection *via* immunosurveillance of the CSF and mount an immune response to prevent progression into the fatal demyelinating disease Progressive Multifocal Leukoencephalopathy (PML) [review e.g., (114)]. During healthy brain surveillance, T cells downregulate their cytolytic granzyme and pro-inflammatory cytokine expression thus preventing autoimmune demyelination (66, 115, 116). It has been suggested that age-related exacerbation of neuroinflammation may disrupt these normal homeostatic functions of immunity and immune surveillance (82).

### T Cells in the Healthy CNS

Recently it has been reported that T cells are beneficial to cognition, including learning and memory (46, 117). Immunodeficient mice, with depleted T cells, display impaired learning and memory which can be rescued by restoration of a normal functioning T cell repertoire (118). These effects are believed to be mediated by CD4^+^ T cells that secrete IL-4, which induces astrocytes to secrete BDNF (119). Myelin-reactive T cells have been shown to play a role in hippocampal neurogenesis and supporting spatial learning (117). However, age-related shifts in the immune system, known as “inflammaging,” cause choroidal T cells to shift toward a pro-inflammatory profile that may be detrimental to cognition (49). Further, T cell infiltration into the temporal lobe of aged mice has been reported to inhibit hippocampal neurogenesis and lead to impairment of spatial learning and memory (51). Recent studies of human AD have shown an influx of CD8^+^ T cells in age-matched control brains, but have not extensively characterized the white matter across the entire lifespan, which we have done here (108).

Our current study did not examine the hippocampus or choroid plexus but instead focused on white matter regions that have previously been associated with both myelin pathology and with cognitive decline observed in normal aging monkeys (19) and humans (75). We specifically were interested in following up on our previous findings that white matter damage and microglial dysfunction in the cingulum bundle of the rhesus monkey were associated with age-related cognitive impairment (19, 35) but now add the observation that T cell infiltration correlates with regional neuroinflammation (activated microglia) as well as cognitive decline.

Very few studies systematically examine the white matter in normal aging so little is known of the function of parenchymal infiltrating T cells. Rodent studies of white matter aging are often inadequate at characterizing the brain late enough in aging to detect the white matter changes we observe in humans and primates (66, 120). Hill et al. found myelin damage comparable to monkeys and humans only in extremely old mice >600 days or 20 months (120). Several reports that examined the normal aging mouse brain have reported an age-related increase in T cells but many report that they are mostly perivascular, meningeal, or choroidal-associated (66, 121). Ritzel and colleagues studied mice out to 22-months of age and report these age-associated T cells promote downregulation of microglial reactivity and encourage a state of surveillance (66). Instead, we suggest T cells that actively infiltrate aging white matter parenchyma may be myelin reactive and could play a detrimental role in allowing or perhaps exacerbating age-related myelin damage and associated cognitive decline. While we have confirmed the age-related increase in perivascular T cells, which may indeed be regulatory, we add that in the aging primate, specifically those exhibiting the most severe cognitive decline, T cells increase in the parenchyma as well. Of note, it is possible that the myelin sheath damage, accumulation of myelin debris, and microglial dysfunction that we observe in our monkeys (19, 35) lead to a more detrimental T cell response in the aging white matter parenchyma of the primate brain, which has a significantly greater proportion of white matter tissue than rodents (122).

### Conclusions

The observations reported here show that T cells infiltrate the aged brain white matter in the absence of infection, damage or blood brain barrier breakdown. Moreover, this infiltration is associated with age-related cognitive impairment. This T cell infiltration into the aging white matter parenchyma constitutes a novel observation that may reflect the mechanism by which cells of the adaptive immune system, during normal aging, contribute to neuroinflammation, white matter pathology and ensuing cognitive impairments. The present data show that in the normal aging primate, T cell infiltration of the brain white matter is likely an active infiltration that correlates with increases in microglia with an inflammatory phenotype. We hypothesize that these infiltrating T cells may be myelin reactive, due to increased exposure to myelin antigens with age, and thus could directly exacerbate myelin pathology in aging. These memory T cells may become activated by the pro-inflammatory neuro-environment (123) and secrete granzymes that may damage the myelin sheath (124). Alternatively, infiltrating T cells may be regulatory and enter in an attempt to moderate neuroinflammation by downregulating microglial reactivity (66). However, given our findings that T cell infiltration is significantly associated with worsening cognitive impairment, we believe that T cells are likely to play a detrimental role in the propagation of age-related neuroinflammation and myelin damage. We suggest that this may constitute a model on which to design more mechanistic studies to examine trafficking routes, antigen-specificity, and inflammatory phenotype of T cells that infiltrate the aging primate brain. Hence these infiltrating T cells offer a new target for interventions aimed at slowing or even reversing age-related neuroinflammation and cognitive decline.

## Data Availability Statement

The raw data supporting the conclusions of this article will be made available by the authors, without undue reservation.

## Ethics Statement

The animal study was reviewed and approved by Boston University Institutional Animal Care and Use Committee (IACUC).

## Author Contributions

Experimental design, execution, and data analysis by KB and DR. Fibrinogen experiments largely carried out by PC. Behavioral data collection and management by TM. Manuscript preparation and editing by all.

## Conflict of Interest

The authors declare that the research was conducted in the absence of any commercial or financial relationships that could be construed as a potential conflict of interest.
